# Genome-wide investigation of the clinical significance and prospective molecular mechanism of minichromosome maintenance protein family genes in patients with Lung Adenocarcinoma

**DOI:** 10.1371/journal.pone.0219467

**Published:** 2019-07-19

**Authors:** Kang Liu, Min Kang, Xiwen Liao, Rensheng Wang

**Affiliations:** 1 Department of Radiation Oncology, The First Affiliated Hospital of Guangxi Medical University, Nanning, Guangxi Zhuang Autonomous Region, People's Republic of China; 2 Department of Hepatobiliary Surgery, The First Affiliated Hospital of Guangxi Medical University, Nanning, Guangxi Zhuang Autonomous Region, People's Republic of China; University of South Alabama Mitchell Cancer Institute, UNITED STATES

## Abstract

Our current study is to identify clinical significance of minichromosome maintenance (MCM) gene expression in Lung Adenocarcinoma (LUAD) using genome-wide RNA sequencing (RNA-seq) dataset and bioinformatics analysis tools. The biological function and potential process for function of the MCM1-10 were identified by multiple bioinformatics analysis tools. Clinical significance and molecular mechanism of the MCM1-10 were investigated by the RNA-seq dataset of LUAD from The Cancer Genome Atlas. Functional assessment substantiated involvement of MCM1-10 in cell cycle progression and DNA replication, and co-expressed with each other. We also observed that the MCM1-10 were dysregulation in LUAD tumor tissues, and may be have diagnostic implications in LUAD. Prognosis analysis in TCGA and KM plotter cohorts suggest that high abundance of *MCM5*, *MCM8* and *MCM4* notably correlated to poor LUAD overall survival. Mechanistic exploration of *MCM4*, *MCM5*, and *MCM8* by gene set enrichment analysis suggests that these genes may influence the LUAD prognosis by regulating the cell cycle, DNA replication and other multiple biological processes and pathways. In comclusion, our study suggests that MCM1-10 can serve as diagnostic biomarkers for LUAD patients. Of them, *MCM4*, *MCM5*, and *MCM8* may act as potential prognostic indicators for LUAD.

## Introduction

Worldwide, Lung cancer is known to have the highest rate of morbidity and mortality worldwide. Current statistics predict 2.1 million new cases and 1.8 million deaths in 2018. One of the areas with the highest incidence is East Asia (China, Japan and South Korea have an incidence of more than 40 / 100,000) [1].The most prevalent kind of lung cancer (about 80%) is non-small cell lung cancer (NSCLC). It can be divided into adenocarcinoma, squamous cell lung cancer and other variety, among which adenocarcinoma is the most common, accounting for more than 50%[2,3].In recent years, despite the continuous progress and development in diagnosis and treatment regimen, the prognosis is still poor, especially for patients with lung adenocarcinoma; the survival rate over a period of 5 years is only 4% - 17%[3–5]. Therefore, it is necessary to actively explore effective tumor biomarkers to predict patients' prognosis and improve survival rate.

Minichromosome maintenance (MCM) genes perform an important function in cell cycle and replication of our genome. This family of genes includes ten members: *SRF* (as know as *MCM1*), *MCM2*, *MCM3*, *MCM4*, *MCM5*, *MCM6*, *MCM7*, *MCM8*, *MCM9* and *MCM10*[6,7].Extensive research have substantiated that they are significantly involved in multiple cancers, and can be regarded as diagnostic and prognostic biomarker[8–12]. However, detailed analysis of the clinical significance of MCM1-10 in LUAD has not been reported, and still needs further exploration. The TCGA database involves large-scale genome sequences to map genomic variants of all human cancers. These sequences are systematically analyzed to find different small mutations of carcinogenic and tumor suppressor genes and understanding the mechanism of cancer cell development. On the above basis new diagnostic and therapeutic methods can be obtained and outlined as the entire new "prevention strategy for cancer"[13].

Our current study aims to investigate the clinical significance and prospective molecular mechanism(s) of MCM1-10 in LUAD using genome-wide RNA sequencing (RNA-seq) dataset and metagenomics analysis tools.

## Methodology

### Data obtained

TCGA database was used as the source for RNA-seq information as well as corresponding clinical parameters of LUAD patients (https://portal.gdc.cancer.gov/,accessed September 06, 2018). To validate the results obtained from the TCGA database, LUAD patients in Kaplan Meier plotter (K-M plotter, http://kmplot.com/analysis/,accessed September 06, 2018)[14]were used as the validation cohort. Since all dataset used in the present study were obtained from open access database, hence, additional ethics committee approval was not necessary.

### Bioinformatics analysis

MCM genes were functionally evaluated using the Database for Annotation, Visualization and Integrated Discovery version 6.8 (DAVID v6.8, https://david.ncifcrf.gov/home.jsp, accessed September 06, 2018) [15,16].We considered p value < 0.05 to be statistically significant. GeneMANIA (http://www.genemania.org/, accessed September 06, 2018) [17]and Search Tool for the Retrieval of Interacting Genes/Proteins (STRING, https://string-db.org/, accessed September 06, 2018) were used to explore gene-gene and protein-protein interactions of MCM1-10 respectively[18–20].

### Co-expression and diagnostic value investigation

Distribution of MCM1-10 between LUAD tumor and matched normal lung tissues were assessed by independent sample t-test. We used Pearson’s correlation coefficient for evaluating the co-expression relationship among the proteins. *Corrplot* package of R platform was used for graphical representation of the data. The distribution analysis of MCM1-10 between normal lung and cancer tissues were carried out by Metabolic gEne RApid Visualizer (MERAV, http://merav.wi.mit.edu/, accessed September 8, 2018) [21]. We used SPSS software to obtain Receiver operating characteristic (ROC) curve to predict the diagnostic values of the MCM1-10 in patients with LUAD.

### Survival analysis

We clustered patients into high and low expression pools based on median values of each MCM gene. We investigated the cooperative effect of MCM genes on survival analysis by taking into consideration those which were significantly associated with LUAD OS. We employed time-dependent ROC curve to assess the precision of the MCM genes in LUAD prognosis prediction. It was constructed using the *survival ROC* package of R platform[22]. Association between prognostic MCM genes and clinical status in LUAD OS was determined through nomogram and joint effect survival analysis.

### Gene set enrichment analysis

We used gene set enrichment analysis (GSEA, http://software.broadinstitute.org/gsea/index.jsp, accessed September 16, 2018)[23] for finding out probable molecular mechanism(s) underlying the role of prognostic MCM genes in LUAD prognosis. The potential mechanisms were explored in the Molecular Signatures Database (MSigDB)[24] of c2 (c2.all.v6.2. symbols) and c5 (c5.all.v6.2.symbols). The significant results of GSEA were identified using the following statistical cutoff values: a nominal *P*-value <0.05 and false discovery rate (FDR) <0.25.

### Statistical analysis

Benjamini–Hochberg algorithm was used to factor for FDR in the GSEA[25]. We used Kaplan-Meier method with log-rank test to plot univariate survival analysis for clinical parameters and MCM genes. These clinicopathological features were found to be significantly involved with LUAD OS (*P* < 0.05). We subjected the factors to multivariate Cox proportional hazards regression model for adjustment and the hazard ratios (HRs) and 95% confidence intervals (CIs) were used to evaluate the proportional risk in different LUAD patients. SPSS software, version 24.0 (IBM Corporation, Armonk, NY, USA) and R 3.3.1 were used to evaluate the statistical analyses. *P*-value <0.05 was considered to be statistically significant.

## Results

### Data source

Our present findings included information from 515 patients having 535 LUAD tumor and 59 adjacent normal lung tissues. A total of 500 LUAD patients with complete prognosis information and RNA-seq data were involved for further survival analysis. We observed significant association between TNM gradation of the tumor and median survival time (MST; P<0.0001;**)**.

### Bioinformatics analysis

**Fig 1:**
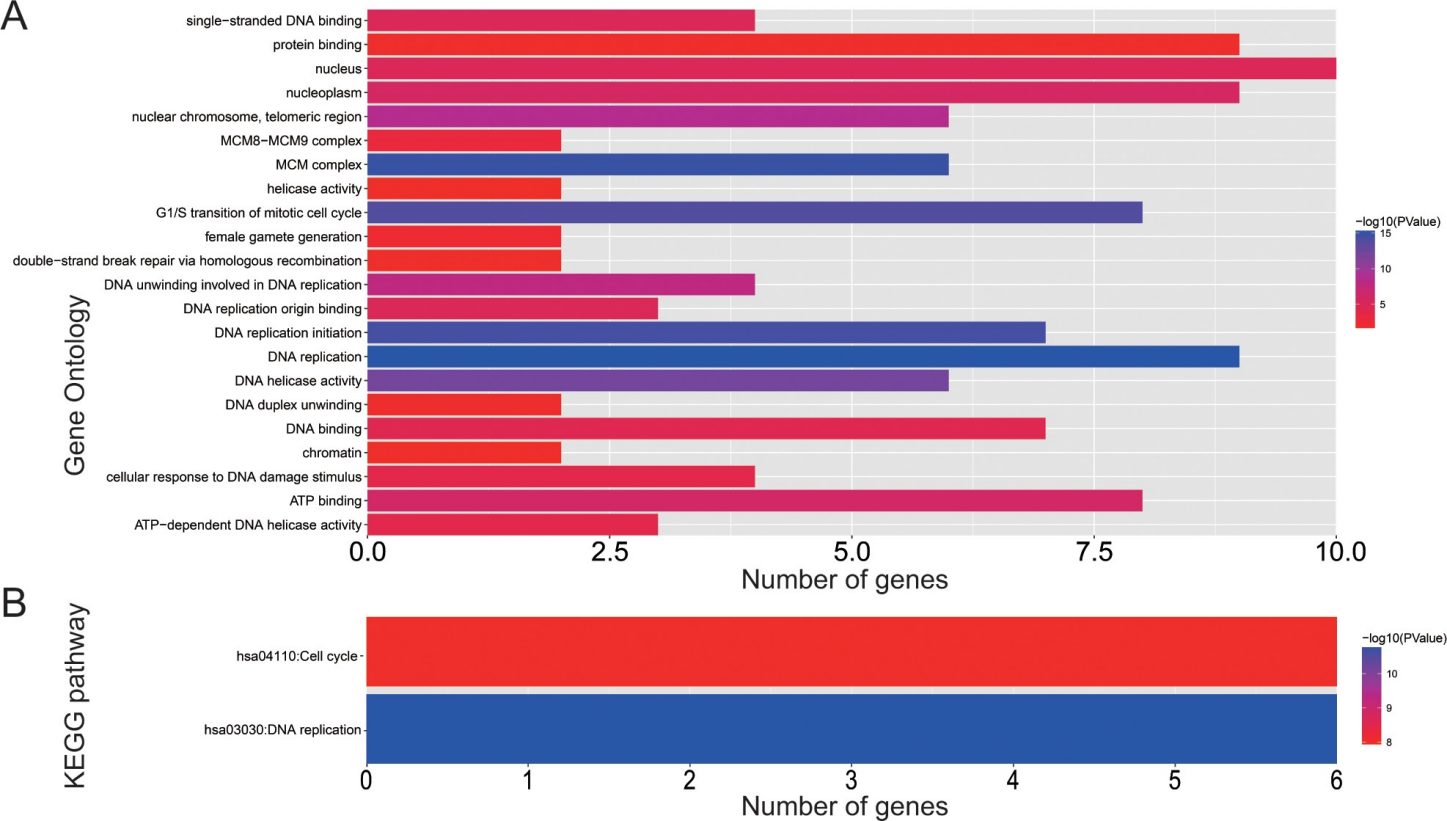
GO term and KEGG analysis of MCM1–10 genes. (A) GO term enrichments of MCM1–10 genes. (**B**) KEGG enrichments of MCM1–10 genes.

**Fig 2:**
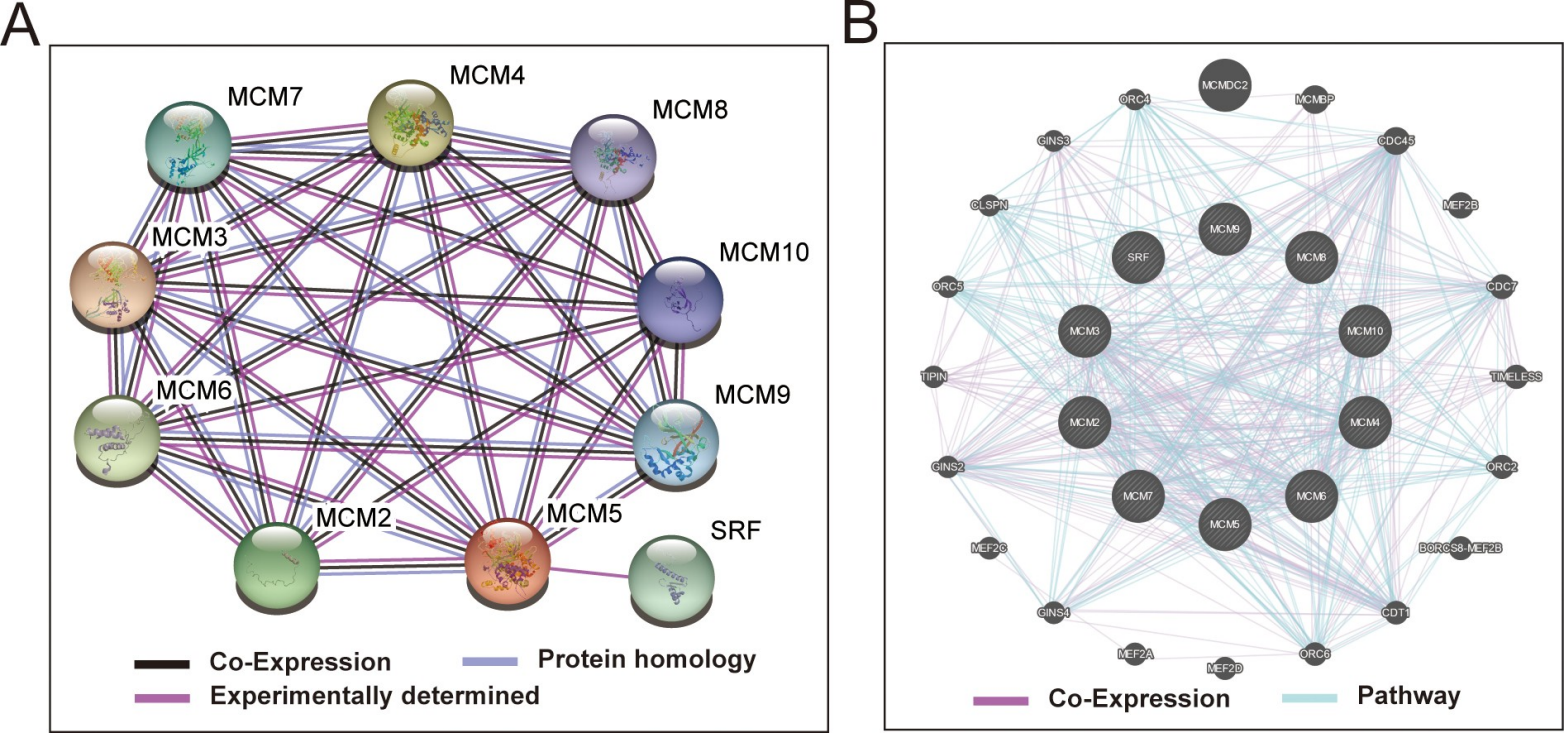
Protein-protein and gene-gene interaction networks of MCM1–10 genes. (A) Protein–protein interaction networks; (B) GeneMANIA interaction networks.

**Fig 3:**
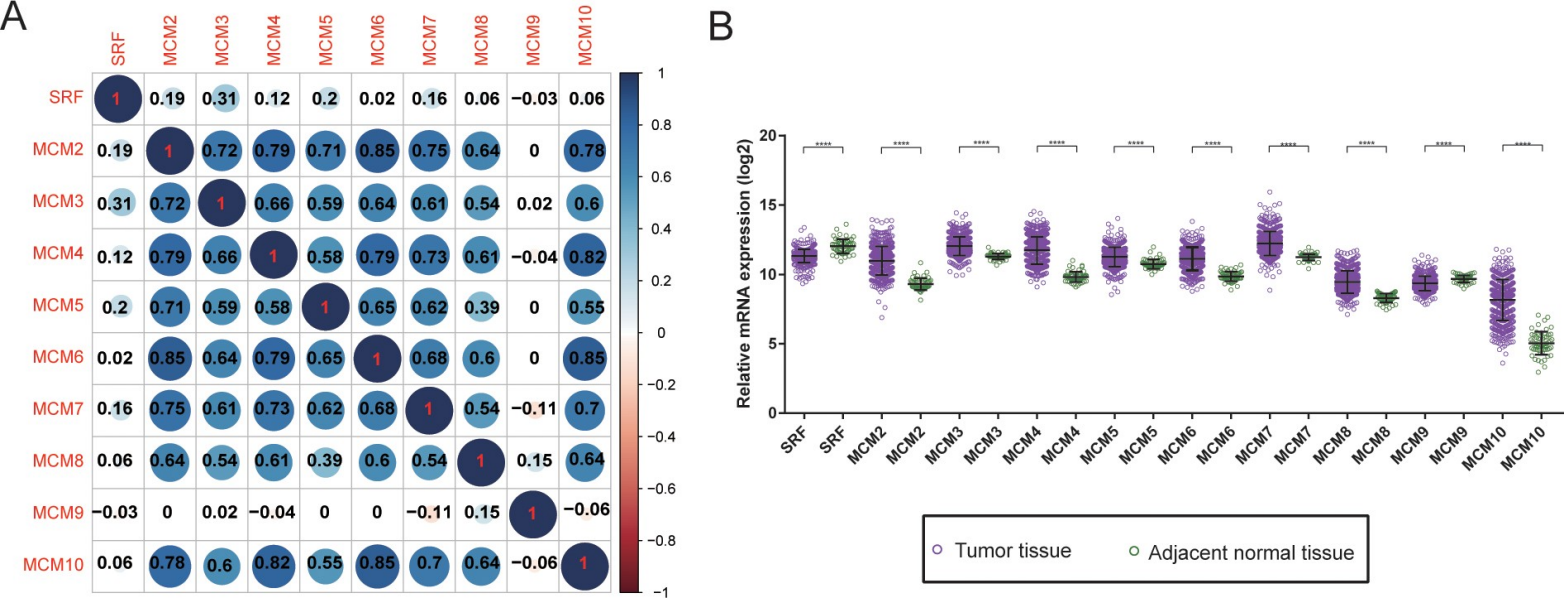
Co-expression heat map and gene expression distribution of MCM genes in TCGA cohort. (A) co-expression heat map of MCM genes in TCGA; (B) gene expression distribution of MCM genes in TCGA.

Biological processes investigation by gene ontology (GO) term **e**nrichment analysis using DAVID v6.8 suggested that these MCM genes were significantly associated with cell cycle progression through G1/S, DNA damage response and DNA replication related biological events namely DNA unwinding involved in DNA replication, replication origin binding, initiation of replication, helicase activity, and DNA duplex unwinding (**Fig 1A**). Moreover, pathway enrichment through Kyoto Encyclopedia of Genes and Genomes (KEGG) using DAVID v6.8 substantiated that these MCM genes were involved in DNA replication and cell cycle functions (**Fig 1B**). Gene-gene as well as protein-protein communication networks established that MCM1-10 exhibit strong homology in their sequences and are co-expressed, which were further corroborated by GeneMANIA and STRING (**Fig 2A and 2B**), respectively. The co-expression relationship of MCM1-10 has also been verified in the LUAD tumor tissues, we observed that these MCM genes also significantly positive co-expressed with each other except MCM9 (**Fig 3A**).

### Differential levels of MCM genes between LUAD tumor and matched normal tissues

**Fig 4:**
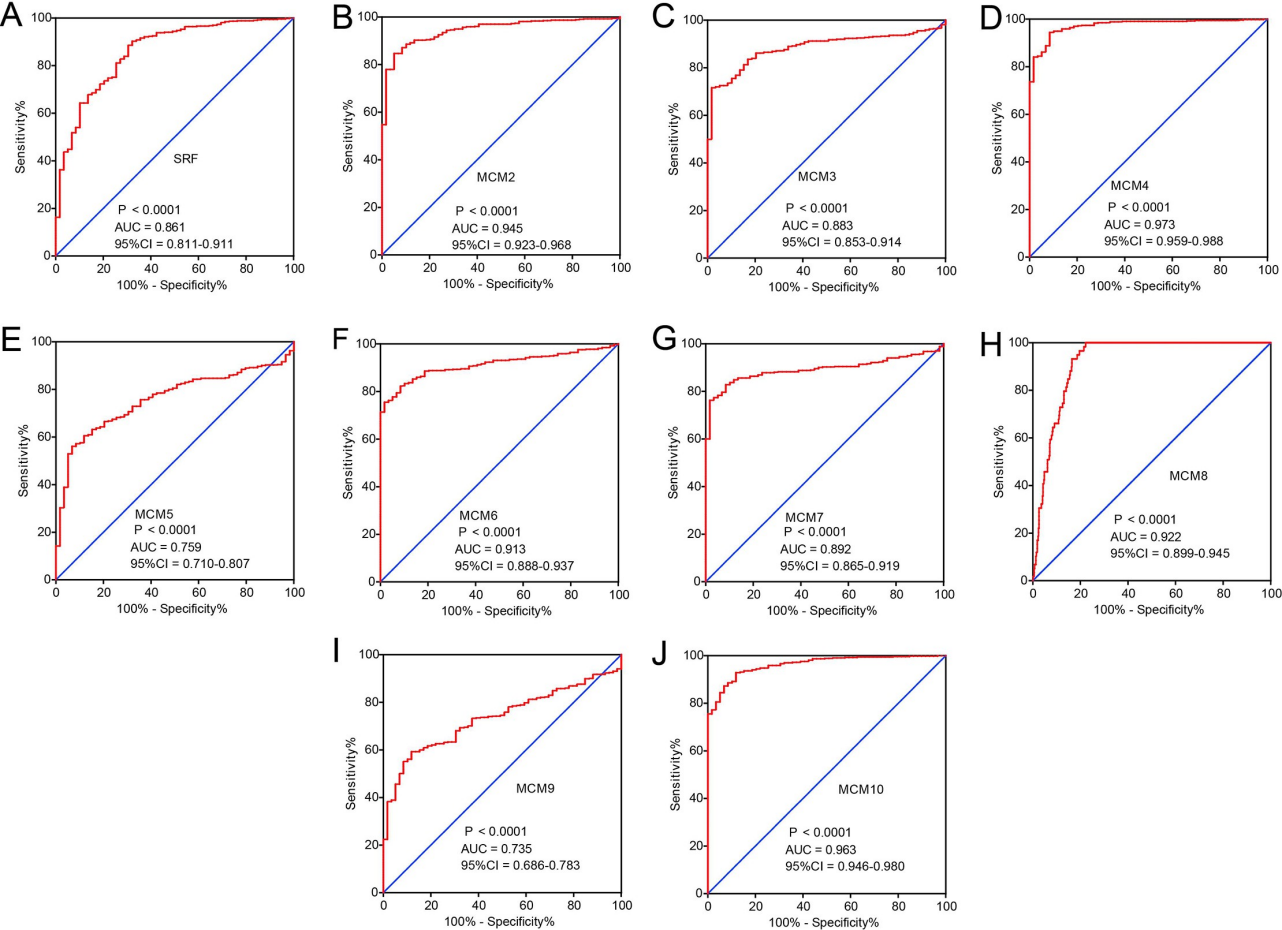
The ROC curves of MCM gens in distinguish LUAD tumor tissue and adjacent normal tissues in TCGA cohort. ROC curves of *MCM1* (A), *MCM2* (B), *MCM3* (C), *MCM4*(D), *MCM5* (E), *MCM6* (F) *MCM7* (G) *MCM8* (H) *MCM9* (I)and *MCM10* (J).

A comparison of the distribution of MCM genes between tumor and adjacent normal lung tissues showed MCM2-8 and MCM10 to be significantly up-regulated in LUAD tumor tissues in TCGA cohorts, whereas the SRF and MCM9 were significantly down-regulated in the same (**Fig 3B**). Distribution of MCM gene expression between lung tumor and adjacent normal tissues using MERAV showed the same trend (**S1A–S1I Fig**). ROC analysis helped to investigate the potential clinical implication of MCM1-10 in differentiating LUAD tumor and matchedt normal lung tissues. The ROC analysis in TCGA LUAD cohort observed all the MCM family members to have high accuracy in distinguishing between LUAD tumors from matched normal lung tissues (the area under the curve [AUC] of the ROC for MCM1-10 rank from 0.735 to 0.973, **Fig 4A–4J**).

### Survival analysis of MCM genes

**Fig 5:**
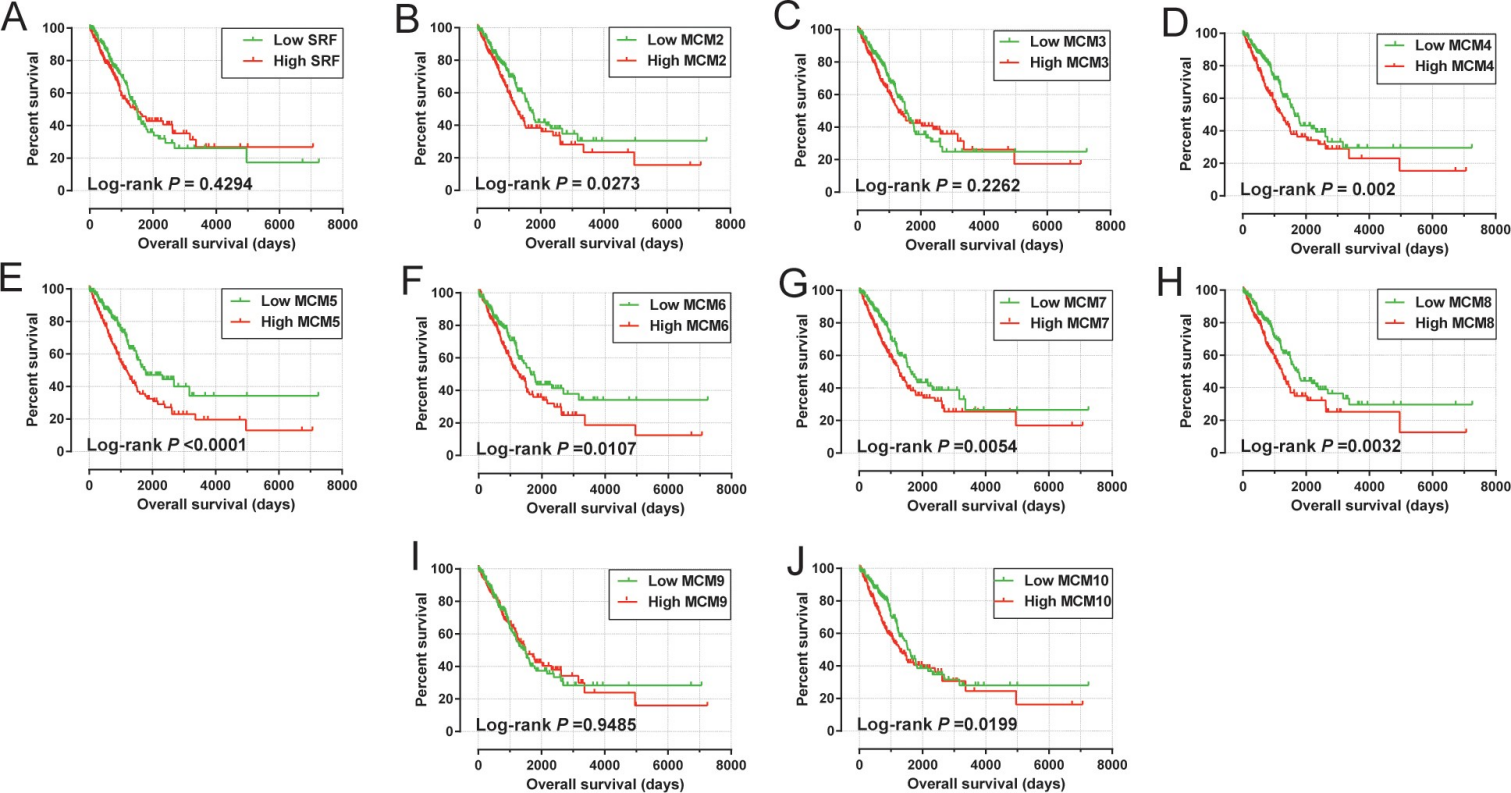
Kaplan–Meier survival curves for MCM gens in TCGA cohort (https://portal.gdc.cancer.gov/). OS stratified by MCM1 (A), MCM2 (B), MCM3 (C), MCM4(D), MCM5 (E), MCM6 (F) MCM7 (G) MCM8 (H) MCM9 (I)and MCM10 (J).

**Fig 6:**
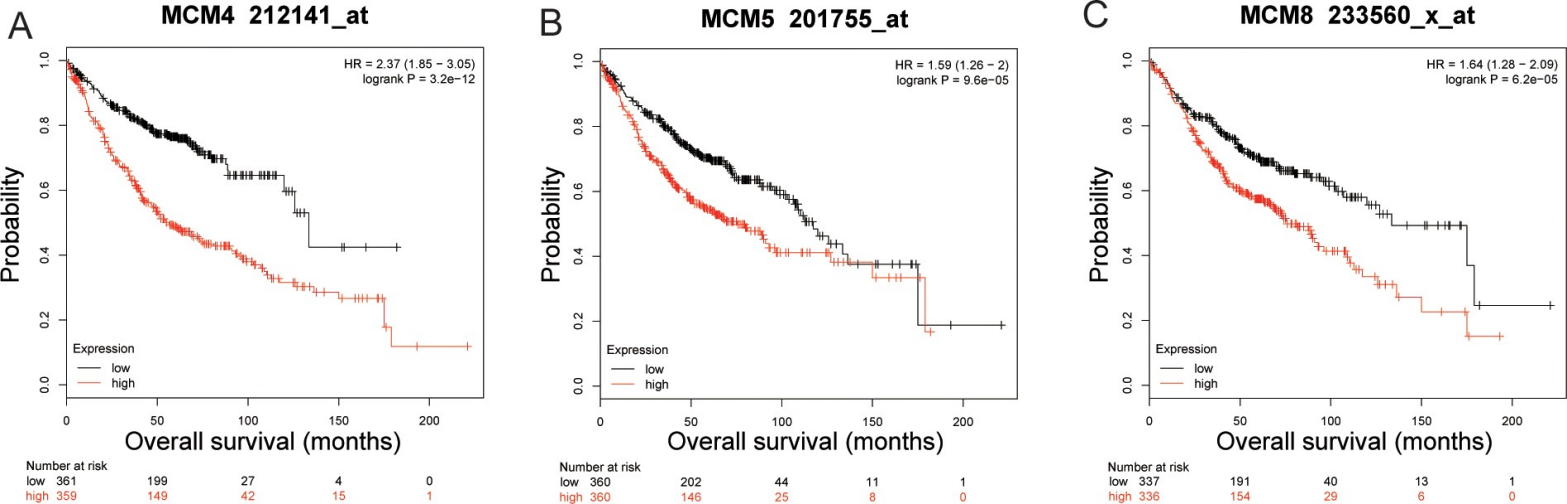
The mRNA expression of MCM4 (A), MCM5 (B), and MCM8 (C) were significantly associated with LUAD OS in patients from the Kaplan Meier plotter web site (http://kmplot.com/analysis/index.php?p=service&cancer=lung).

Multivariate Cox proportional hazards regression model suggest that elevated levels of MCM4 (adjusted *P* = 0.044, adjusted HR = 1.362, 95%CI = 1.008–1.842, **Table 1, Fig 5A–5J**), MCM5 (adjusted *P* = 0.003, adjusted HR = 1.579, 95%CI = 1.163–2.143, **Table 1, Fig 5A–5J**) as well as MCM8 (adjusted *P* = 0.030, adjusted HR = 1.391, 95%CI = 1.003–1.873, **Table 1, Fig 5A–5J**) significantly increased risk of death for patients with LUAD. Prognostic values of MCM4, MCM5 and MCM8 in the KM plotter website further indicated that high expression of these MCM genes were associated with poor LUAD OS (**Fig 6A–6C**).

**Table 1:** Prognostic values of MCM genes expression in LUAD cohort of TCGA.

**Genes**	**Expression level**	**Event/Total**	**MST**	**Crude HR(95%CI)**	**Crude P**	**Adjusted HR(95%CI)**	**Adjusted P&**
SRF	Low	88/250	1498	1		1	
	High	94/250	1421	1.125(0.840–1.505)	0.43	1.091(0.812–1.467)	0.563
MCM2	Low	80/250	1653	1		1	
	High	102/250	1258	1.390(1.036–1.864)	0.028	1.196(0.887–1.613)	0.24
MCM3	Low	84/250	1501	1		1	
	High	98/250	1293	1.197(0.894–1.603)	0.227	1.178(0.877–1.583)	0.276
MCM4	Low	77/250	1653	1		1	
	High	105/250	1229	1.585(1.180–2.128)	0.002	1.362(1.008–1.842)	0.044
MCM5	Low	69/250	1790	1		1	
	High	113/250	1171	1.842(1.365–2.486)	< 0.0001	1.579(1.163–2.143)	0.003
MCM6	Low	77/250	1653	1		1	
	High	105/250	1288	1.464(1.090–1.966)	0.011	1.294(0.960-.745)	0.091
MCM7	Low	75/250	1622	1		1	
	High	107/250	1265	1.516(1.128–2.038)	0.006	1.226(0.904–1.664)	0.191
MCM8	Low	83/250	1725	1		1	
	High	99/250	1235	1.552(1.156–2.085)	0.003	1.391(1.003–1.873)	0.030
MCM9	Low	95/250	1498	1		1	
	High	87/250	1499	0.990(0.740–1.325)	0.948	1.089(0.810–1.464)	0.573
MCM10	Low	79/250	1600	1		1	
	High	103/250	1288	1.414(1.055–1.897)	0.021	1.237(0.917–1.669)	0.163

**Notes:** &Adjusted for tumor stage. MST, median survival time; HR, hazard ratio; CI, confidence interval; NA, not available; MCM, minichromosome maintenance; TCGA, The Cancer Genome Atlas.

**Fig 7:**
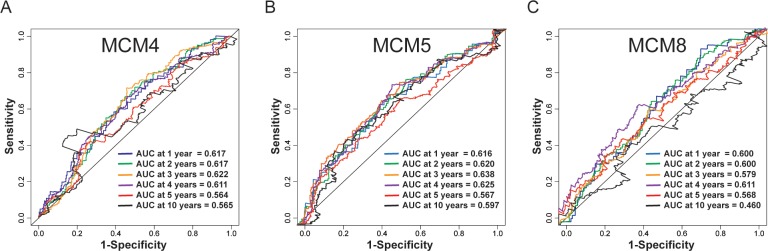
ROC curve for predicting survival in LUAD patients by the risk score MCM4 (A),MCM5 (B), and MCM8 (C).

**Fig 8:**
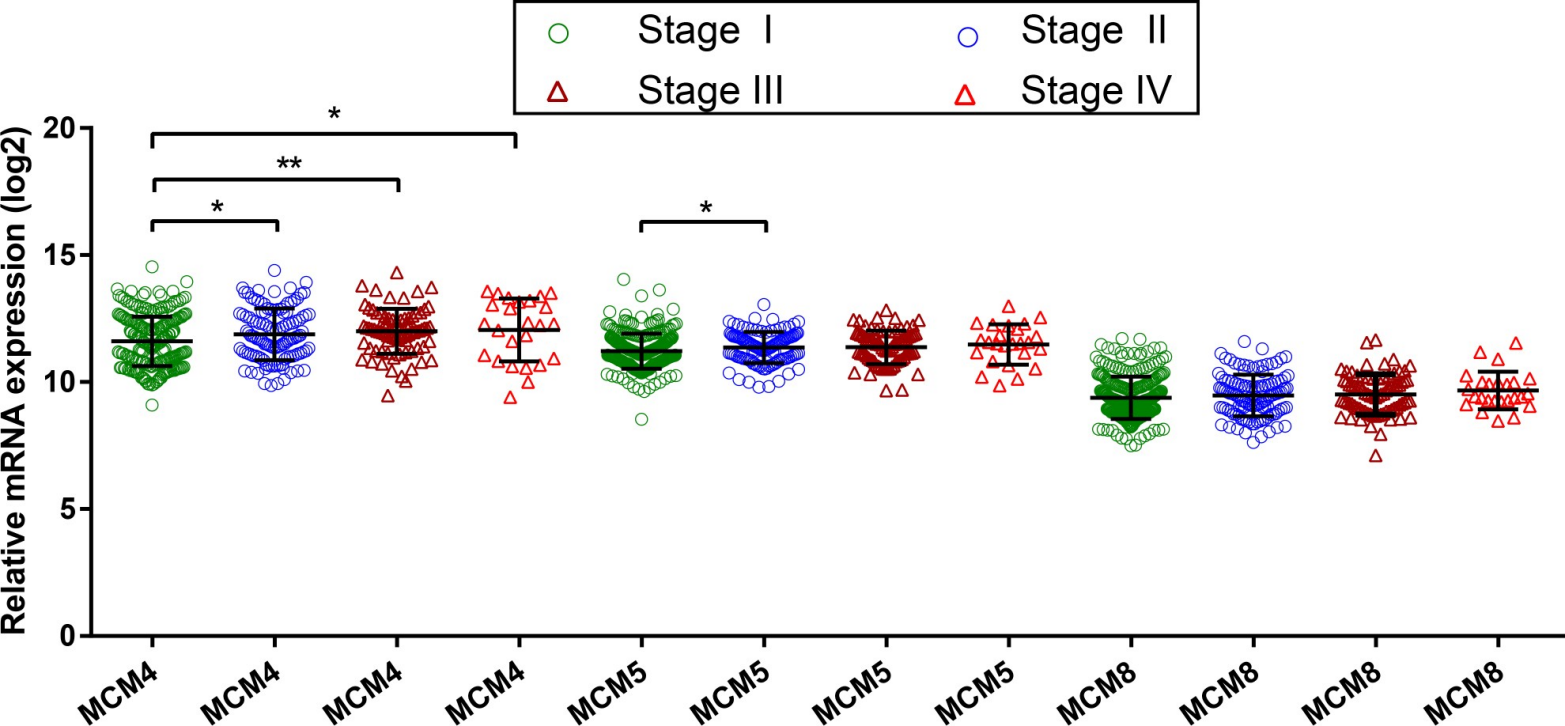
Gene expression distribution of MCM genes in TCGA.

**Fig 9:**
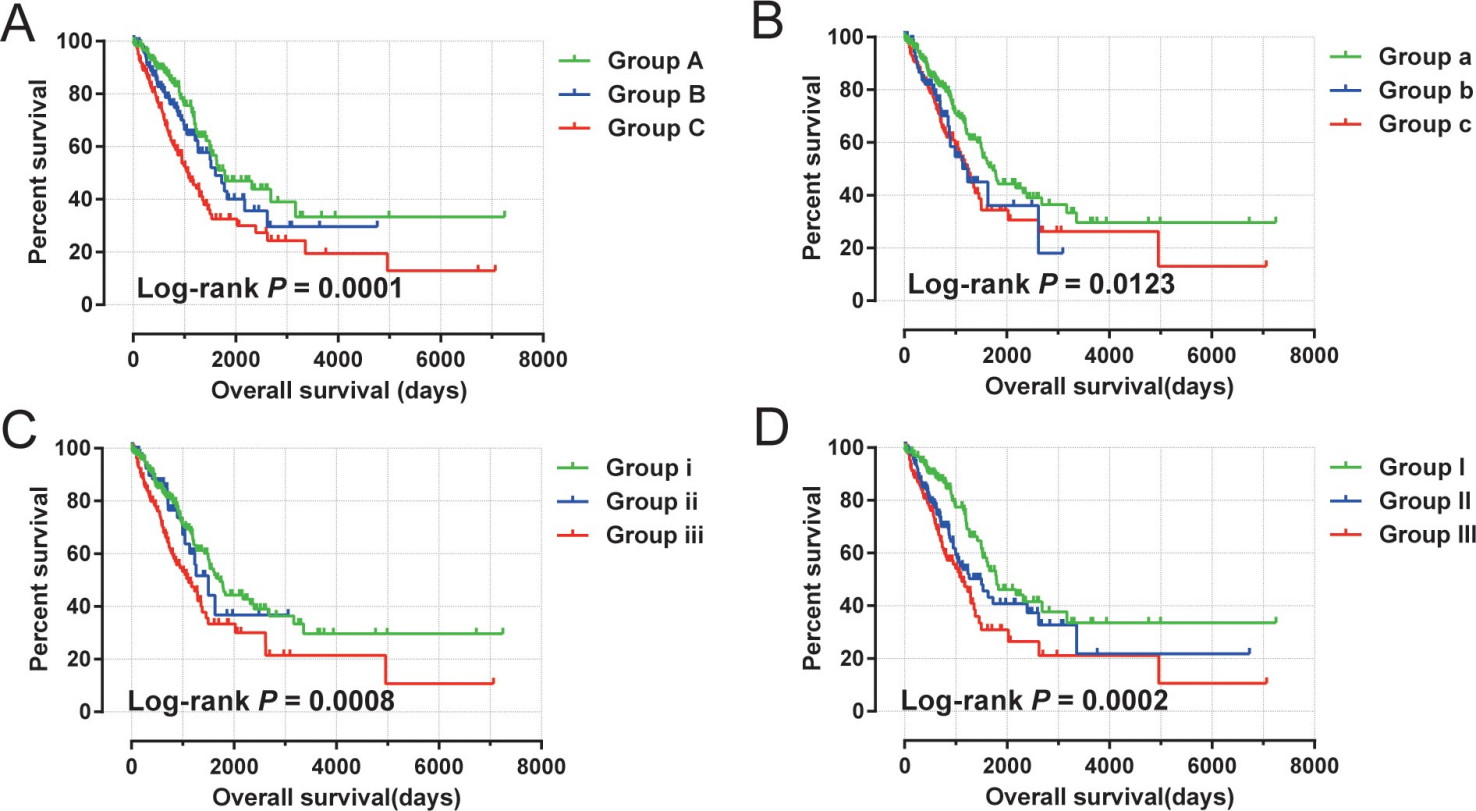
Kaplan–Meier survival curve for joint effects analysis of *MCM4*, *MCM5* and *MCM8* genes in LUAD patients. (A) Joint effects analysis of *MCM4* and *MCM5*;(B) Joint effects analysis of *MCM4* and *MCM8*; (C) Joint effects analysis of *MCM5* and *MCM8*;(D) Joint effects analysis of *MCM4*, *MCM5* and *MCM8*.

**Fig 10:**
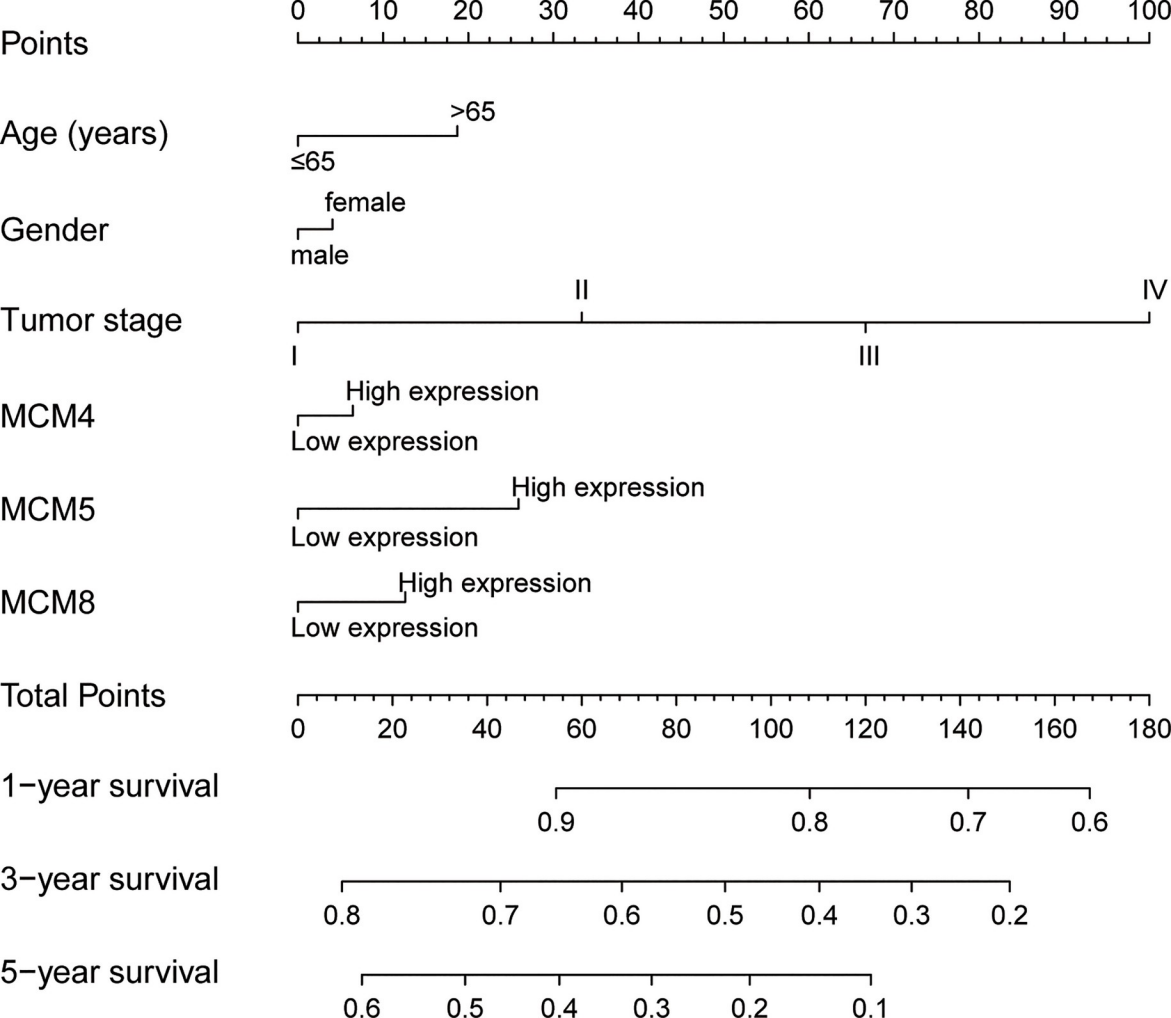
Nomogram for predicting the 1-,3- and 5-year event (death) with risk score and clinical information.

Time-dependent ROC analysis (using *survival ROC* package of R) helped to assess accuracy of MCM gene in LUAD prognosis prediction. We observed that MCM4 and MCM5 have some predictive value in predicting the prognosis of LUAD (**Fig 7A–7C**). In addition, we also evaluated MCM4, MCM5 and MCM8 in LUAD progression, and demonstrated significant upregulation of MCM4 and MCM5 in advanced stage of LUAD (**Fig 8**). Furthermore, we also explored the joint effect survival analysis among MCM4, MCM5 and MCM8 genes (**Table 2**), and found the combination of high expressions of these genes significantly predicted a worse OS than the combination of low MCM expressions. The difference was also significantly pronounced than single high MCM expression group (**Table 3, Fig 9A–9D**). The nomogram also suggested that the MCM4, MCM5 and MCM8 genes could be used as prognostic indicators for LUAD (**Fig 10**).

**Table 2:** Grouping information of joint survival analysis.

**Genes**	**Group**	**Combination**
MCM4+MCM5		
	Group A	low MCM4 + low MCM5
	Group B	high MCM4 +low MCM5; low MCM4 +high MCM5
	Group C	high MCM4 + high MCM5
MCM4+MCM8		
	Group a	low MCM4 + low MCM8
	Group b	high MCM4 +low MCM8; low MCM4 +high MCM8
	Group c	high MCM4 + high MCM8
MCM5+MCM8		
	Group i	low MCM5 + low MCM8
	Group ii	high MCM5 +low MCM8; low MCM5 +high MCM8
	Group iii	high MCM5 + high MCM8
MCM4+MCM5+MCM8		
	Group I	low MCM4 + low MCM5 + low MCM8
	Group II	high MCM4 + low MCM5 + low MCM8; low MCM4 + high MCM5 + low MCM8; low MCM4 + low MCM5 + high MCM8; high MCM4 + high MCM5 + low MCM8; low MCM4 + high MCM5 + high MCM8; high MCM4 + low MCM5 + high MCM8
	Group III	high MCM4 + high MCM5 + high MCM8

**Notes:** MCM, minichromosome maintenance.

**Table 3:** Joint effects survival analysis of clinical factors and the DEMs’ signature risk score with OS in LUAD patients.

**Gene combination**	**Groups**	**Event/Total**	**MST**	**Crude HR(95%CI)**	**Crude P**	**Adjusted HR(95%CI)**	**Adjusted P&**
MCM4+MCM5							
	Group A	50/180	1790	1		1	
	Group B	46/140	1600	1.341(0.898–2.003)	1.151	1.203(0.797–1.816)	0.378
	Group C	86/180	1073	2.069(1.459–2.935)	<0.0001	1.680(1.175–2.402)	0.004
MCM4+MCM8							
	Group a	83/250	1725	1		1	
	Group b	25/74	1235	1.475(0.940–2.316)	0.091	1.52(0.968–2.388)	0.069
	Group c	74/176	1258	1.580(1.152–2.166)	0.005	1.351(0.981–1.860)	0.066
MCM5+MCM8							
	Group i	83/250	1725	1		1	
	Group ii	24/84	1501	1.094(0.692–1.728)	0.7	1.071(0.677–1.694)	0.77
	Group iii	75/166	1115	1.788(1.306–2.449)	<0.0001	1.541(1.120–2.120)	0.008
MCM4+MCM5+MCM8							
	Group I	52/176	1790	1		1	
	Group II	69/195	1501	1.617(1.126–2.322)	0.009	1.541(1.065–2.230)	0.022
	Group III	61/129	1115	2.152(1.483–3.122)	<0.0001	1.766(1.206–2.585)	0.003

**Notes:** &Adjusted for tumor stage. MST, median survival time; HR, hazard ratio; CI, confidence interval; NA, not available;MCM, minichromosome maintenance; LUAD, lung adenocarcinoma.

### GSEA

**Fig 11:**
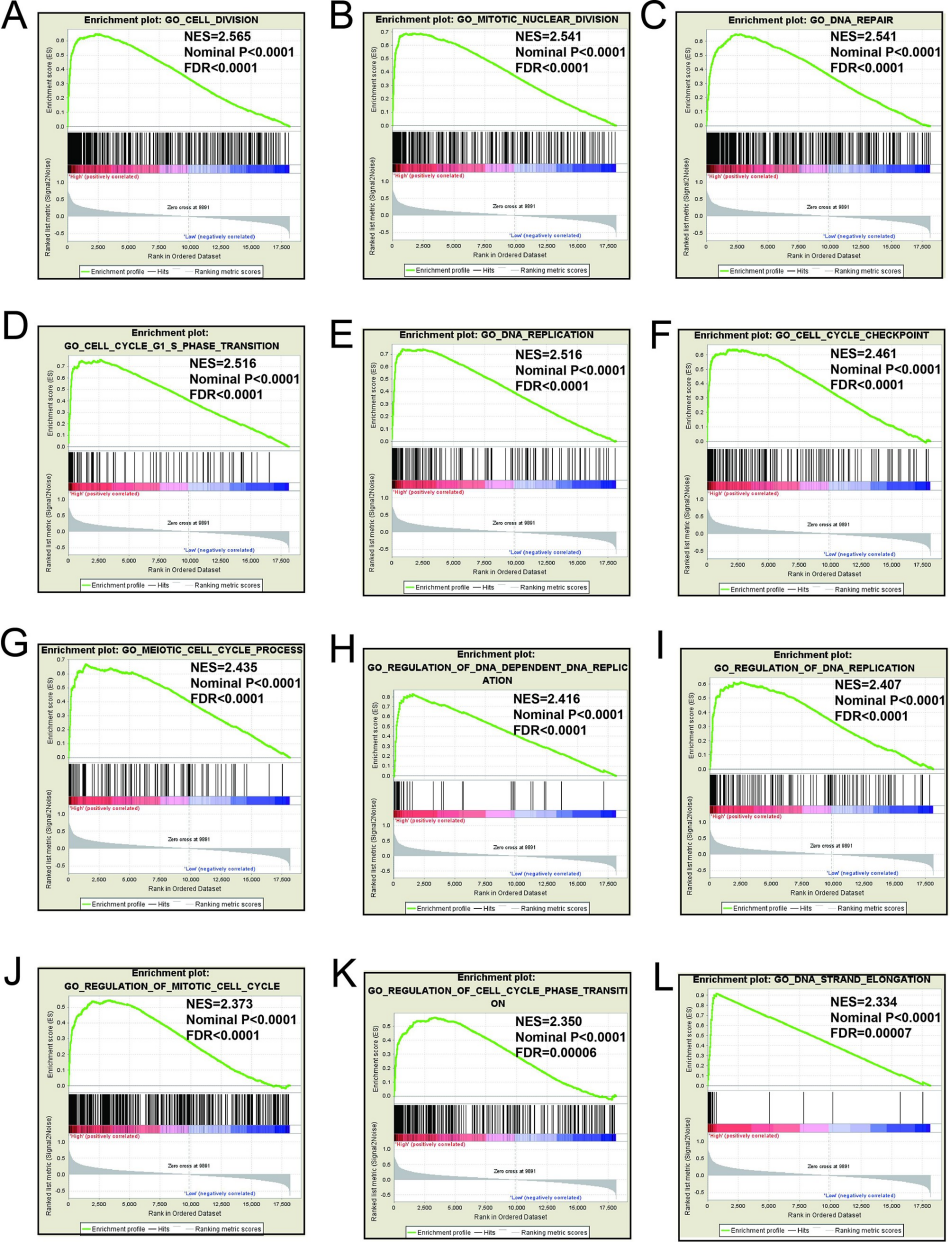
GSEA results of c5 reference gene sets for high MCM4 expression groups. (A)Cell division(B)Nuclear division(C)DNA repair(D)Cell cycle G1 S phase transition(E)DNA replication(F)Cell cycle checkpoint(G)Meiotic cell cycle process(H)Regulation of DNA dependent(I)Regulation of DNA replication(J)Regulation of mitotic cell cycle(K)Regulation of cell cycle phase transition(L)DNA strand elongation.

**Fig 12:**
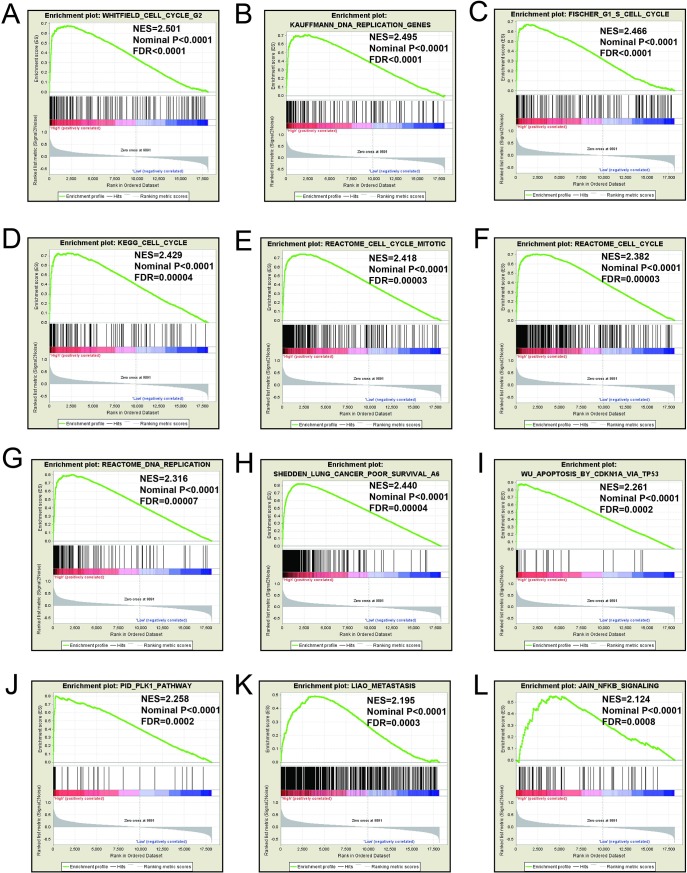
GSEA results of c2 reference gene sets for high MCM4 expression groups. (A)WHITFIELD cell cycle G2(B)KAUFFMANN DNA replication genes(C)FISCHER G1 S cell cycle(D)KEGG cell cycle(E)REACTOME cell cycle mitotic(F)Reactome cell cycle(G)REACTOME DNA replicatrion (H)SHEDDEN LUNG CANCER poor survival (I)Apoptosis by CDKN1A VIA TP53 (J)PID PLK1 pathway (K)LIAO metastasis (L)JIAN NFKB signaling.

**Fig 13:**
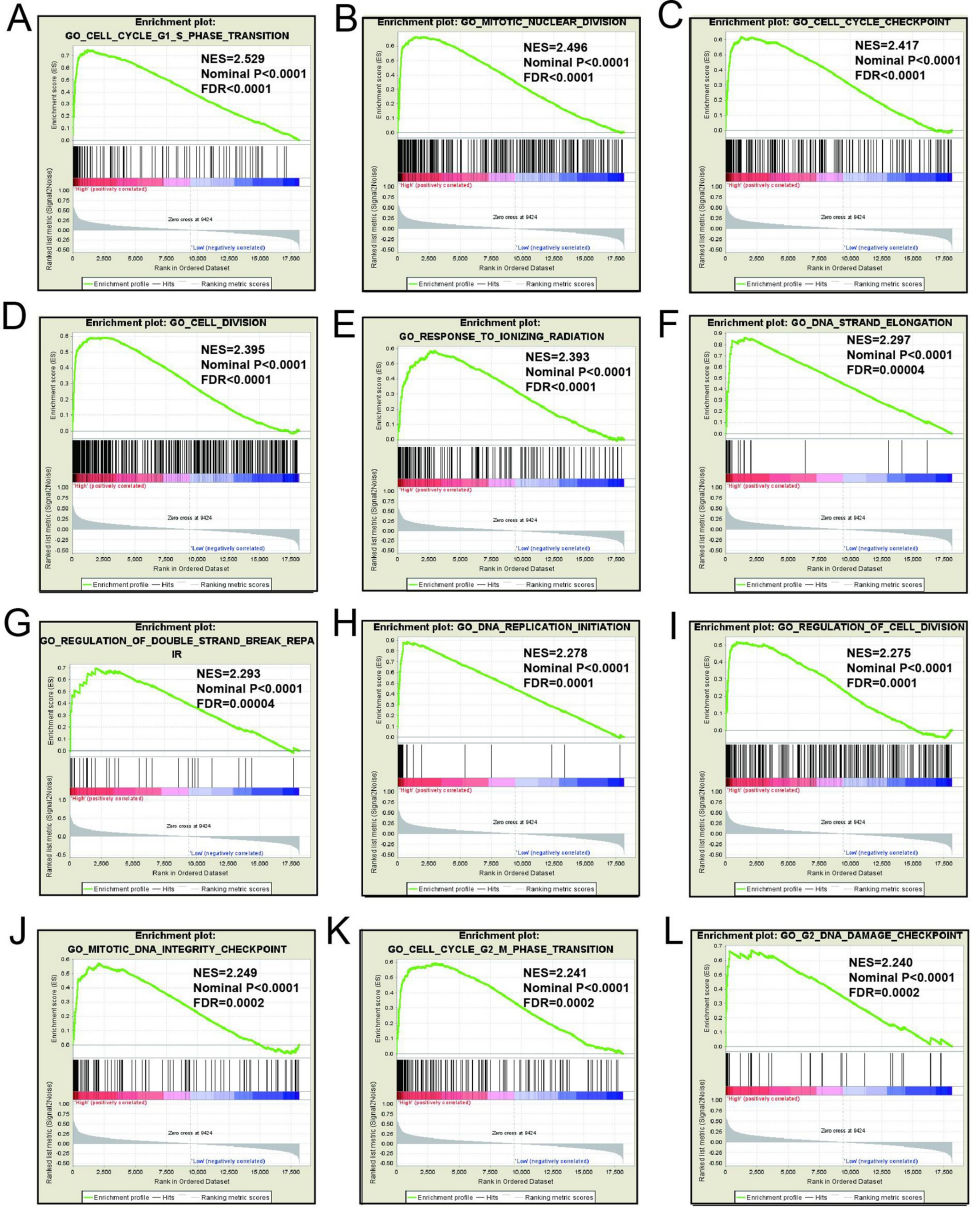
GSEA results of c5 reference gene sets for high MCM5 expression groups. (A) Cell cycle G1 S phase transition (B)Nuclear division(C) Cell cycle checkpoint (D) Cell division (E)Response to ionizing radiation(F)DNA strand elongation(G)Regulation of double strand break repair(H)DNA replication initiation(I) Regulation of cell division (J)Mitotic DNA integrity checkpoint (K) Cell cycle G2 M phase transition (L) G2 DNA demage checkpoint.

**Fig 14:**
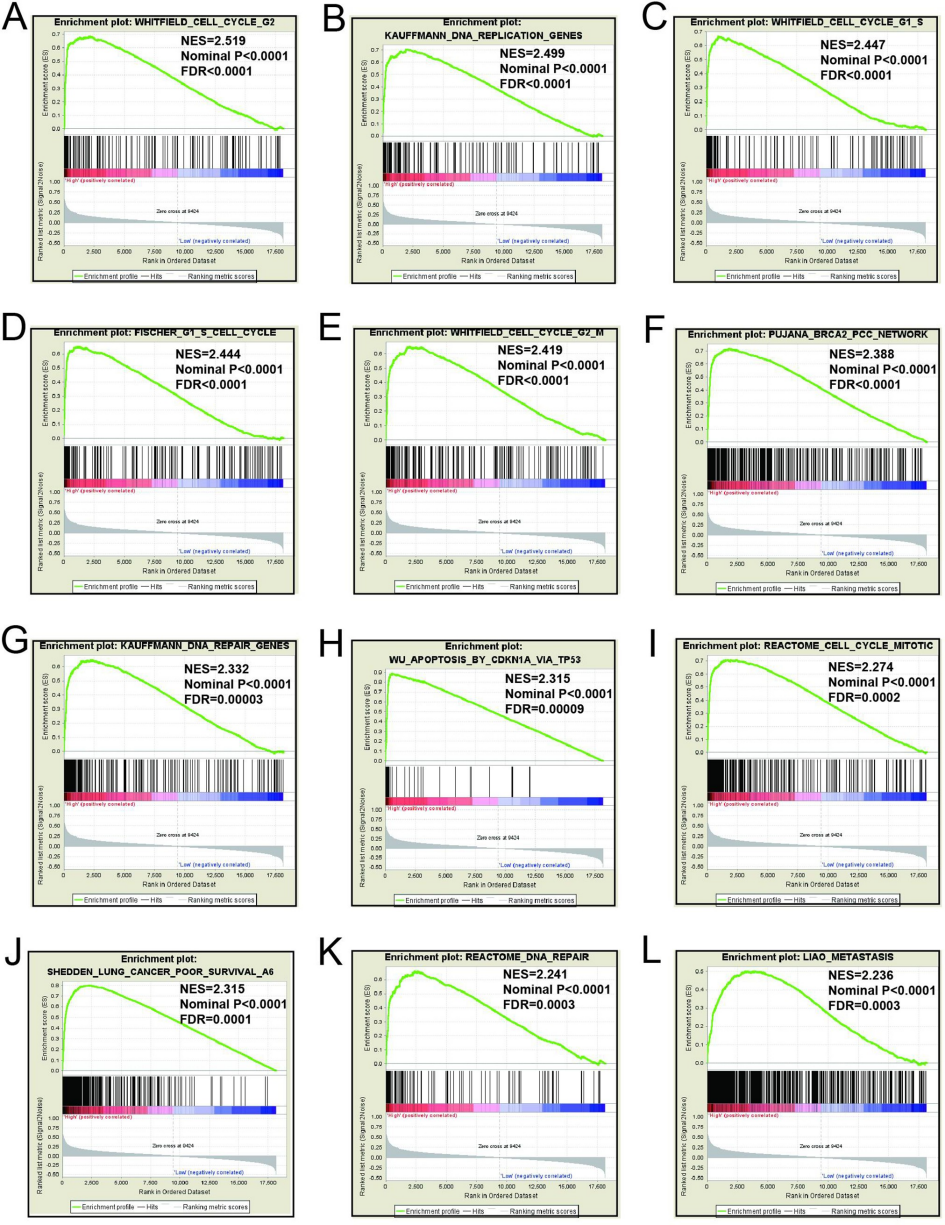
GSEA results of c2 reference gene sets for high MCM5 expression groups. (A)WHITFIELD cell cycle G2(B)KAUFFMANN DNA replication genes(C) WHITFIELD cell cycle G1 S(D) FISCHER G1 S cell cycle (E)WHITFIELD cell cycle G2 M(F)PUJANA BRCA2 pcc network(G) KAUFFMANN DNA repair genes(H)WU Apoptosis by CDKN1A VIA TP53 (I) REACTOME cell cycle mitotic(J) SHEDDEN LUNG CANCER poor survival(K) REACTOME DNA repair (L)LIAO metastasis.

**Fig 15:**
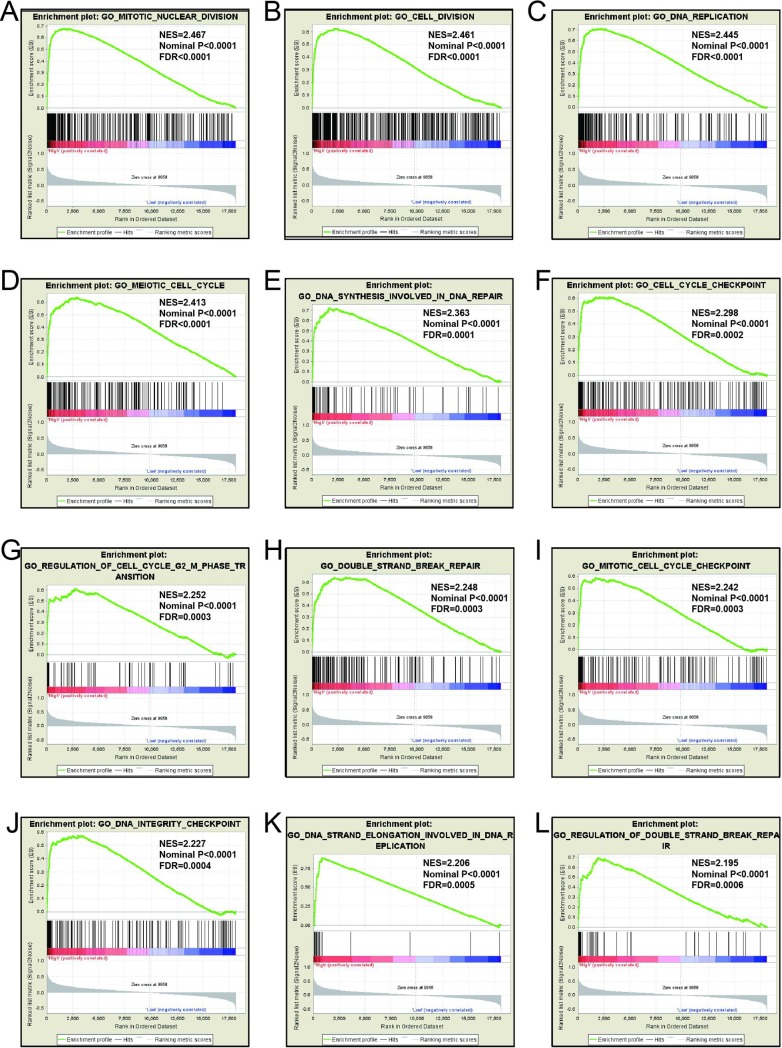
GSEA results of c5 reference gene sets for high MCM8 expression groups. (A) Meiotic nuclear division(B)Cell division(C)DNA replication(D)Meiotic Cell cycle(E)DNA synthesis involved in DNA repair(F)Cell cycle checkpoint(G)Regulation of cell cycle G2 M phase transition(H)Double strand break repair(I)Meiotic cell cycle checkpoint (J)DNA ingegrity checkpoint (K) DNA strand elongation (L)Regulation of double strand break repair.

**Fig 16:**
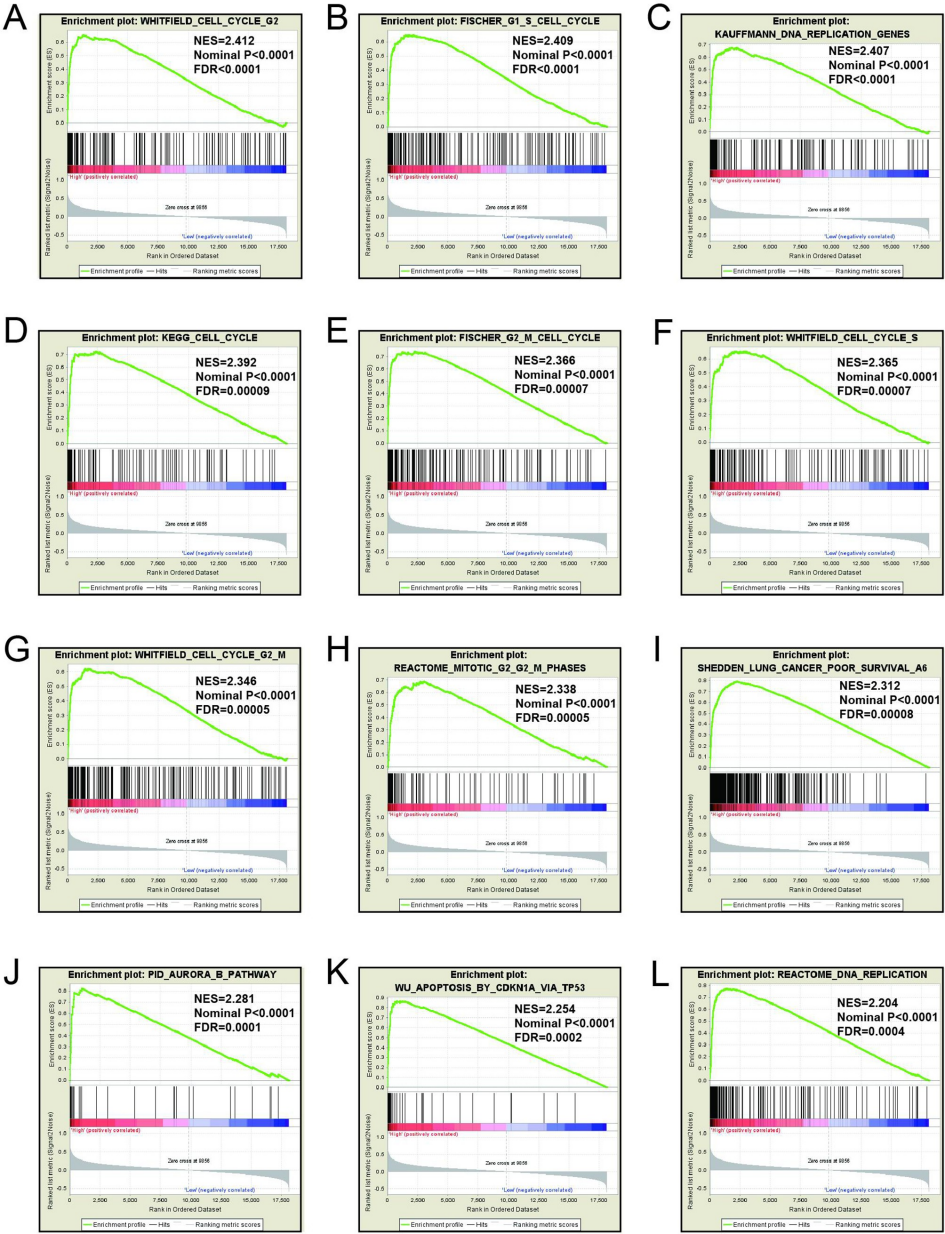
GSEA results of c2 reference gene sets for high MCM8 expression groups. (A)WHITFIELD cell cycle G2(B) FISCHER G1 S cell cycle (C) KAUFFMANN DNA replication genes(D)KEGG cell cycle(E) FISCHER G2 M cell cycle (F) WHITFIELD cell cycle S (G) WHITFIELD cell cycle G2 M(H) REACTOME mitotic G2 M phases(I) SHEDDEN LUNG CANCER poor survival A6 (J)PID AURORA B pathway (K) Apoptosis by CDKN1A VIA TP53 (L) REACTOME DNA replication.

Mechanistic exploration of *MCM4*, *MCM5*, and *MCM8 by GSEA* showed that these genes may influence the LUAD prognosis by regulating cell cycle and DNA replication related biological events, cell and mitotic nuclear division, apoptosis by CDKN1A via *TP53*, as well as various other biological processes and pathways (**Fig 11A–11L; Fig 12A–12L; Fig 13A–13L; Fig 14A–14L; Fig 15A–15L; Fig 16A–16L**). Furthermore, the C2 enrichment also indicated that these genes to be relevantly involved with poor survival statistics of lung cancer (**Fig 12H, Fig 14J, Fig 16I**). C5 enrichment of MCM4 suggest that except for cell cycle and DNA replication, high MCM4 expression can also influence DNA repair (**Fig 11)**, whereas the C2 enrichment suggested high MCM4 to regulate PLK1 pathway, metastasis, and NF-KB pathway (**Fig 12**). C5 enrichment of MCM5 indicate that high MCM5 expression also regulates the DNA damage checkpoint (**Fig 13)**, wherein the C2 enrichment was suggestive that high MCM5 takes part in AURORA pathway, metastasis, and DNA repair (**Fig 14**). C5 enrichment of MCM8 indicate that high MCM8 expression modulates the DNA double strand break repair function (**Fig 13)**, whereas the C2 enrichment speaks of MCM8 to be associated with AURORA pathway (**Fig 14**).

## Discussion

The MCM protein family, which is encoded by MCM genes, is a highly conserved family with closely related members. It is composed of six subunits, including MCM2, MCM3, MCM4 (CDC21), MCM5 (CDC46), MCM6 (Mis5) and MCM7 (CDC47), and exhibit the helicase activity of replicating DNA.[26]MCM8, MCM9, MCM1 and MCM10 are also found in multicellular organisms and play crucial functions in DNA replication.[7,27]Our bioinformatics analysis also demonstrated that MCM1-10 is involved in cell cycle and replication of DNA. Co-expression analysis substantiated that production of MCM1-10 were strongly co-regulated both in genetics and proteomic levels, as well as in LUAD tumor tissues.

Numerous studies have observed dysregulation of MCM1–10 in multiple cancers, and serve as potential diagnostic biomarkers. Previous reports showed increased expression of MCM2 in colorectal cancer (CRC) tissues could be adopted as diagnostic biomarker.[28]The MCM3 immune-histochemical staining can be adopted as biomarker for oral squamous cell carcinoma early detection.[29] MCM4 has been opined to participate in the tumorigenesis of esophageal cancer.[30,31]Different cancers like pancreaticobiliary malignancy (PBC), [32,33]esophageal cancer [11]and cervical cancer (CC)[34] have been studied in the light of MCM5 as a diagnostic marker. *MCM6* may serve as potential biomarkers in patients with hepatocellular carcinoma(HCC)[22]. In addition, MCM7 has been helpful in early detection of gastric cancer (GC) [35], and altered diagnosis between malignant mesothelioma (MMCS) and reactive mesothelial cells (MCS)[36]. Deregulation of MCM2–7 can be potential biomarkers in meningioma tumor tissues[37].Consistent with the previous findings, our present work observed deregulations of MCM1–10 genes in LUAD cancer tissues which can be serve as potential diagnostic biomarkers.

In current study, we identified and verified *MCM4*, *MCM5*, and *MCM8* levels to be significantly correlated to LUAD OS in TCGA and KM plotter cohorts. High expression of these genes significantly involved in LUADs poor clinical outcome. Joint effects survival analysis suggested that the combined high expressions of MCM significantly predicted a worse OS than the combination of both low MCM expressions. Similar to the results of previous studies, there are several reports that high MCM genes were involved with poor OS in multiple cancers. Multiple reports have substantiated that the elevated MCM2 production was associated with a poor prognosis in GC patients,[38,39] lung cancer (LC),[40] and urothelial bladder carcinomas (BUCS).[41]High MCM3 was significantly associated with OS in patients with astrocytoma.[42] It was previously observed that the high MCM4 expression predicts detrimental prognosis in patients with esophageal adenocarcinoma (EAC).[31] Study suggested that MCM4 profiling could potentially be used to predict response to treatment and prognosis in laryngeal squamous cell carcinoma (SCC)[43].Upregulation of MCM5 was also observed in LC and CC, and patients with high MCM 5 expression faced increased morbidity.[44,45] Previous studies observed that the elevated status of MCM6 was proportional to unfavorable prognosis in patients with non-small cell lung carcinoma(NSCLC),[46]mantle cell lymphoma(MCL),[47]and endometrioid endometrial adenocarcinoma(EEA)[48]in both the protein and mRNA level.MCM7 expression identified by immune-histochemical staining also observed the similar results in NSCLC.[49,50]These previous studies proved that the MCM7 gene can serve as a prognostic biomarker, and high amounts of the protein in these tumors show significant correlation with an unfavorable OS thus making MCM7 a significant prognostic biomarker. Literature review also suggests prognostic values of MCM1-10 in LUAD, and indicates that MCM genes may be considered to be oncogenes in multiple cancer types. At present, there are few studies on MCM8.Study has shown that MCM2, 4, 8 and 10 overexpression is associated with shorter overall survival for PC. Further multivariate analysis showed that MCM8 is an independent prognostic factor. However, our results still need further *in vivo* and *in vitro* experimental verifications.

GSEA for different expression levels of *MCM4*, *MCM5* and *MCM8* indicated that significant correlation of the genes with lung cancer progression and survival. Potential molecular mechanisms of *MCM4*, *MCM5* and *MCM8* in LUAD prognosis may involve different biological functions of the cell cycle, DNA replication, DNA repair, NF-kB, apoptosis via *TP53* and AURORA B pathway. As we all know, MCM genes are pivotal for DNA replication and cell cycle process.[7]Study showed that enhanced NF-κB expression as a prognostic predictor is positively correlated with poor survival outcome of NSCLC patients.[51]MCM4 may affect the prognosis of lung cancer via NF-κB pathway.[52] Aurora kinase B regulates cell mitosis by adjusting the chromosomal passenger complex. MCM genes may regulate cell mitosis via AURORA B pathway.[53]However, this conjecture should be further validated.

The present study comes with its own limitations which require explanation. The RNA-seq dataset and clinical parameters included in our work were downloaded from TCGA website, and these data were imperfect and incomplete. Therefore, a perfect multivariate Cox proportional hazards regression model analysis which considered all LUAD relative prognostic clinical parameters was beyond the scope of the study. Second, since our current study only focusses on LUAD, the diagnostic and prognostic values in the lung cancer remains to be further explored and the molecular mechanism is yet to be further confirmed.

In spite of the above limitations, our study has implicated MCM4, MCM5 and MCM8 in prognosis of patients with LUAD, and also unraveled their molecular mechanism(s) in LUAD through GSEA. In addition, we also observed the dysregulation of MCM genes also can function as potential diagnostic biomarkers in patients with LUAD. Once these results are verified in the sample proteome, these MCM genes may have underlying clinical values in LUAD. However, our findings still need to be verified in another prospective study with larger cohort and design.

## Conclusions

In current study, we found that MCM1-10 have underlying diagnostic values in individuals suffering from LUAD. *MCM4*, *MCM5* and *MCM8* may be considered as potential prognostic biomarkers in patients with LUAD as observed from survival analysis results. Combination of *MCM4*, *MCM5* and *MCM8* could also be adopted as independent indicators for LUAD OS prediction as observed through joint effects survival analysis. The potential molecular mechanism of *MCM4*, *MCM5* and *MCM8* in LUAD prognosis may correlate to cell cycle, DNA repair and DNA replication associated biological functions and signaling pathways. However, these prospective molecular mechanisms and clinical significance still need *in vivo*, *in vitro* experiments and clinical trial validations.

## Supporting information

S1 Fig MERAV boxplots for MCM genes expression in normal lung tissue and lung cancer tissue: *MCM1* (A), *MCM2* (B), *MCM3* (C), *MCM4*(D), *MCM5* (E), *MCM6* (F) *MCM7* (G) *MCM8* (H) *MCM9* (I)and *MCM10* (J).
(TIF)
