# Supplementary figures and images for: Genome-wide investigation of the clinical significance and prospective molecular mechanism of minichromosome maintenance protein family genes in patients with Lung Adenocarcinoma
Source: PLoS One. 2019 Jul 19;14(7):e0219467. doi: 10.1371/journal.pone.0219467 (PMC6641114; doi:10.1371/journal.pone.0219467)

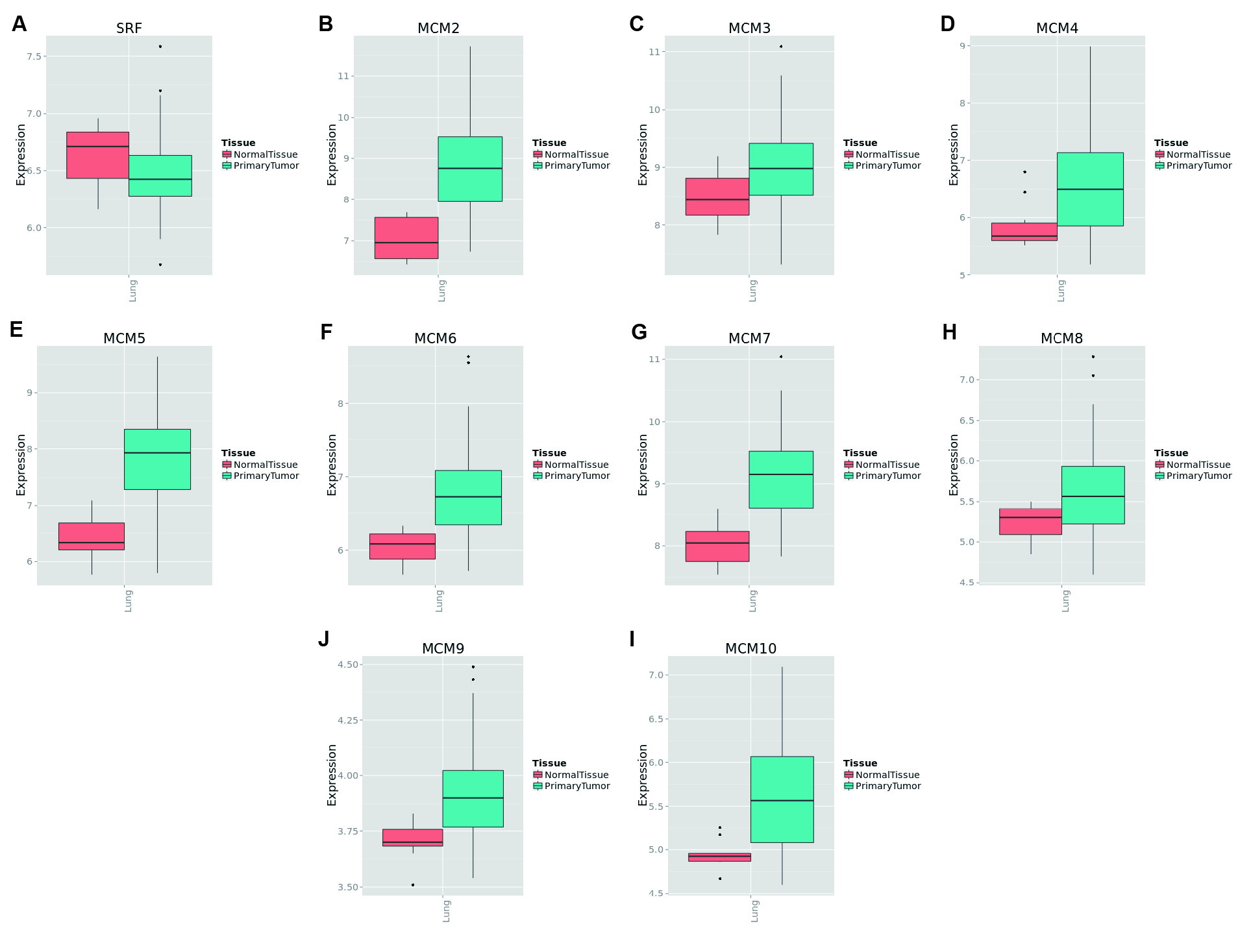

Supplement: S1 Fig — MERAV boxplots for MCM genes expression in normal lung tissue and lung cancer tissue: MCM1 (A), MCM2 (B), MCM3 (C), MCM4(D), MCM5 (E), MCM6 (F) MCM7 (G) MCM8 (H) MCM9 (I)and MCM10 (J). (TIF) [file pone.0219467.s001.tif]
